# Immunization with a *Borrelia burgdorferi* BB0172-Derived Peptide Protects Mice against Lyme Disease

**DOI:** 10.1371/journal.pone.0088245

**Published:** 2014-02-05

**Authors:** Christina M. Small, Dharani K. Ajithdoss, Aline Rodrigues Hoffmann, Waithaka Mwangi, Maria D. Esteve-Gassent

**Affiliations:** Department of Veterinary Pathobiology, Texas A & M University, College Station, Texas, United States of America; University of North Dakota School of Medicine and Health Sciences, United States of America

## Abstract

Lyme disease is the most prevalent arthropod borne disease in the US and it is caused by the bacterial spirochete *Borrelia burgdorferi* (Bb), which is acquired through the bite of an infected *Ixodes* tick. Vaccine development efforts focused on the von Willebrand factor A domain of the borrelial protein BB0172 from which four peptides (A, B, C and D) were synthesized and conjugated to Keyhole Limpet Hemocyanin, formulated in Titer Max® adjuvant and used to immunize C3H/HeN mice subcutaneously at days 0, 14 and 21. Sera were collected to evaluate antibody responses and some mice were sacrificed for histopathology to evaluate vaccine safety. Twenty-eight days post-priming, protection was evaluated by needle inoculation of half the mice in each group with 10^3^ Bb/mouse, whereas the rest were challenged with 10^5^Bb/mouse. Eight weeks post-priming, another four groups of similarly immunized mice were challenged using infected ticks. In both experiments, twenty-one days post-challenge, the mice were sacrificed to determine antibody responses, bacterial burdens and conduct histopathology. Results showed that only mice immunized with peptide B were protected against challenge with *Bb*. In addition, compared to the other the treatment groups, peptide B-immunized mice showed very limited inflammation in the heart and joint tissues. Peptide B-specific antibody titers peaked at 8 weeks post-priming and surprisingly, the anti-peptide B antibodies did not cross-react with *Bb* lysates. These findings strongly suggest that peptide B is a promising candidate for the development of a new DIVA vaccine (Differentiate between Infected and Vaccinated Animals) for protection against Lyme disease.

## Introduction

Lyme disease (LD) is the most prevalent arthropod-borne infection in the United States with 30,831 cases of LD reported to the Centers for Disease Control and Prevention (CDC) in 2012. A significant increase in the number of reported cases has been observed in the past few years, classifying LD as a re-emerging infection. *Borrelia burgdorferi,* the causative agent of Lyme disease, is transmitted to humans through the bite of infected *Ixodes* ticks [1]-[4]. This pathogen is maintained in nature through a very complex enzootic cycle in which small mammals and birds serve as reservoirs [5]–[7]. This pathogen is accidentally transmitted to humans and companion animals where it causes disease. The ability of this spirochetal pathogen to colonize mammals is dependent on its ability to rapidly alter gene expression in response to highly disparate environmental signals following transmission from infected ticks [8]–[13]. Consequently, a lot of interest has been devoted to the study of proteins differentially expressed in the tick and the mammalian host as a way to identify potential targets for vaccine development. One of the first targets identified using this approach was the borrelial outer surface protein A (OspA) which was the target in the only licensed human Lyme vaccine, LYMErix (SmithKline Beecham) [14]. In the arthropod tick, the OspA protein is expressed by *B. burgdorferi*, adhering to the tick receptor for OspA (TROSPA) located in the tick mid-gut [15]. Upon tick feeding, OspA is down regulated allowing the bacteria to migrate from the tick mid-gut into the salivary glands and from there into the mammalian host [15]–[17]. Taking this into account, the OspA-based vaccine induced high antibody levels in laboratory animals as well as in humans and consequently conferred protection by blocking the transmission of *B. burgdorferi* from the tick to the mammalian host [18]–[21]. Despite the fact that this vaccine showed good protection in phase III human clinical trials, the company voluntarily discontinued the distribution of this vaccine [14], [22]–[24]. This was due to a number of reasons including a significant reduction in the vaccine demand, the appearance of adverse reaction to the vaccine, the complicated immunization protocol with periodic boosts to maintain high antibody titers and age limitations [14], [23], [25], [26]. This vaccine formulation has been used to develop vaccines administered to wild life (small rodents in particular) to lower *B. burgdorferi* burden in the mammalian reservoirs and the tick vectors, thus reducing the risk for human infection [27]–[31]. In addition, the OspA-based vaccine has been used in veterinary medicine for some time (Nobivac® Lyme from Merk Animal Health; LymeVax® formulated by Fort Dodge and Recombitek® Lyme y Merial) to prevent Lyme disease in dogs [32]–[37]. Unfortunately there is no Lyme vaccine currently available for use in humans and horses.

Other differentially expressed proteins such as BBA52, OspC, BBK32 and DbpA, have been evaluated as potential vaccine targets [38]–[43]. However, none of these have been tested in human or veterinary clinical trials. Nevertheless, these target proteins are not optimal vaccines for differentiating infected from vaccinated animals (DIVA vaccines) since both immunized and infected animals respond to these antigens [44]–[47].

In our study, we have selected the chromosomally encoded membrane-associated protein BB0172 of *B. burgdorferi* to develop a DIVA vaccine. We have previously shown that BB0172 [48] inserts into the Borrelia outer membrane and through its von Willebrand Factor A domain (vWFA) binds to the human integrin α_3_β_1_. BB0172 is expressed only when shifting *B. burgdorferi* cultures growing at room temperature with a pH of 7.6 (unfed tick conditions) to 37°C at a pH of 6.8 (fed tick conditions). In addition, BB0172 is not expressed in cultures adapted to either of the conditions and furthermore is not recognized by serum from infected animals nor animals immunized with the full length protein [48]. Thus, a conserved domain in the vWFA-domain of BB0172 could be an excellent candidate for developing a DIVA vaccine due to the highly conserved nature of BB0172 among *B. burgdorferi* sensu lato complex genospecies which cause LD in Europe and the US [48]. In this study, we designed a series of short peptides from the vWFA domain of BB0172 and conjugated them to KLH as potential vaccine candidates. We immunized C3H/HeN mice with each one of the peptides following conventional immunization protocol. Our first goal was to identify the most antigenic peptide, therefore, safety of each one of the peptides was evaluated as well as the protective response they induced in the murine model of Lyme disease. Our second goal was to determine the potential of these peptides to protect against Lyme disease in the murine model, using the tick challenge as the natural way of disease transmission, and elucidate the role of antibodies and T-cells in protection against Lyme disease.

## Results

### Identification of potential vaccine targets from the BB0172 antigen

**Figure 1:**
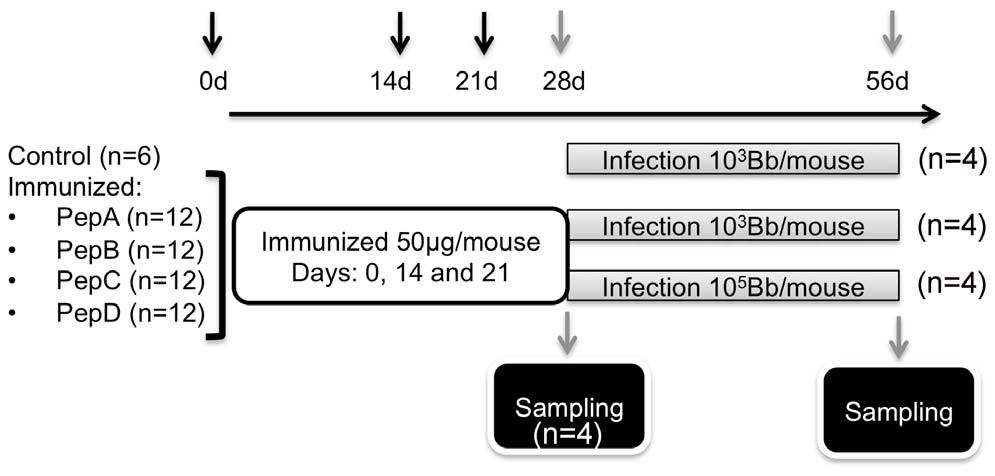
Schematic representation of the target identification phase. C3H/HeN mice were immunized with peptides derived from the VWFA domain of BB0172 (A, B, C and D) conjugated to KLH and administered at 50 µg/mouse with equal volume of TiterMax® Gold (Sigma-Aldrich) at days 0, 14, and 21. Four weeks post-priming, 4 mice per treatment were sampled to evaluate vaccine safety and antibody levels to each one of the peptides used. The other eight mice were infected with either 10^3^ (n = 4) or 10^5^ (n = 4) spirochetes/mouse. Four weeks post-challenge, mice were euthanized and blood collected to determine antibody levels. Tissues were sampled to determine bacterial burden by growth and qPCR as well as to determine any pathology by histology.

The *B. burgdorferi* chromosomally encoded BB0172 protein has been shown by our laboratory to be a membrane protein containing a VWFA domain exposed to the extracellular milieu [48]. In our efforts to obtain specific antibodies against this protein we observed that BALB/c mice or C3H/HeN mice could not raise specific antibodies to the full-length BB0172 protein (Esteve-Gassent, personal observation). After analyzing the amino acid sequence of the vWFA-domain in BB0172, peptides A, B, C and D with B-epitope qualities were designed and conjugated to KLH. Following immunization of groups of mice at days 0, 14 and 21 (Fig. 1), immune protection was tested by challenging the mice 28 days post-priming by needle inoculation of some mice with the low dose of (10×ID_50_) and some mice with the high dose of (1000×ID_50_) borrelial cells/mouse. Twenty-one days post-infection, the mice were euthanized and blood and tissues were collected to evaluate antibody responses, bacterial load and histopathology. *B. burgdorferi* was recovered from tissues (skin, spleen, inguinal lymph node, bladder, heart and joint) from all treatment groups except from tissues collected from mice immunized with pepB and challenged with 10 × ID_50_ (Table 1). Evaluation of bacterial burden in the tissues from pepB vaccinees by q-PCR revealed low to undetectable infection levels (data not shown).

**Table 1:** Peptide B protects after needle inoculation of 10×ID_50_ in the murine model of Lyme disease.

	No. of tissues positive/No. of tissues tested							
Strain and dose	Skin	Spleen	Lymph node	Bladder	Heart	Joint	All sites	No. animals infected/ No. animals tested
**Control**								
10^3^ spirochetes/mouse	4/4	4/4	4/4	4/4	4/4	4/4	24/24	4/4
**PepA**								
10^3^ spirochetes/mouse	4/4	4/4	4/4	4/4	4/4	4/4	24/24	4/4
10^5^ spirochetes/mouse	4/4	4/4	4/4	4/4	4/4	4/4	24/24	4/4
**PepB**								
10^3^ spirochetes/mouse	0/4	0/4	0/4	0/4	0/4	0/4	0/24	0/4***
10^5^ spirochetes/mouse	4/4	4/4	4/4	4/4	4/4	4/4	24/24	4/4
**PepC**								
10^3^ spirochetes/mouse	4/4	4/4	4/4	4/4	4/4	4/4	24/24	4/4
10^5^ spirochetes/mouse	4/4	4/4	4/4	4/4	4/4	4/4	24/24	4/4
**PepD**								
10^3^ spirochetes/mouse	4/4	4/4	4/4	4/4	4/4	4/4	24/24	4/4
10^5^ spirochetes/mouse	4/4	4/4	4/4	4/4	4/4	4/4	24/24	4/4

*** Denotes statistically significant differences (*P* value < 0.001) when compared with the control group.

**Figure 2:**
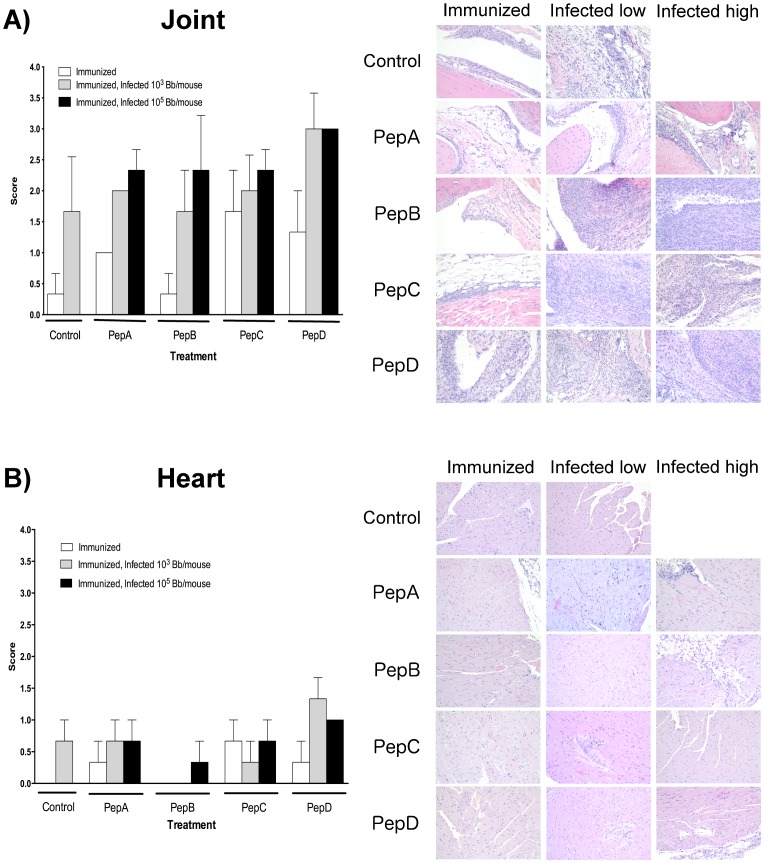
Representative histological images of the average level of inflammation observed in each treatment group (control, pepA, pepB, pepC, and pepD) after immunization and/or infection with the low (10^3^ spirochetes/mouse) or high (10^5^ spirochetes/mouse) doses in the tibiotarsal joint (A) and the heart (B). Tissues were histologically evaluated at four weeks post priming, as well as four weeks post needle inoculation. Average scores for areas of inflammation were classified as 0 =  none; 1 =  minimal; 2 =  mild; 3 =  moderate; 4 =  severe. Peptide B induces minimal inflammation in hearts and tibiotarsal joints after administration in the mouse model for Lyme disease. Of all the peptides evaluated after immunization, only peptide B showed inflammation comparable to the negative control group in both heart and joints. Similar results were observed after infection with low doses of *B. burgdorferi*. Images were captured using an Olympus BX41 microscope at 200X magnification. Average ± SD are presented in the graphs.

Vaccine or *B. burgdorferi*-induced inflammation in joints and heart was evaluated by histological analysis of these tissues collected 4 weeks post-priming and 4 weeks post-infection, respectively (Fig. 2). PepB-vaccinees developed a minimal inflammation in the tibiotarsal joint similar to the background inflammation observed in the control non-immunized group (Fig. 2A). Peptides C and D vaccinated mice had moderate to severe inflammation in the tibiotarsal joint after immunization (Fig. 2A). Severe inflammation was observed in mice challenged with 1000 × ID_50_ with mice immunized with pepD having the most severe tibiotarsal joint inflammation among the groups tested. Histological evaluation of the heart revealed that pepB vaccinees had no signs of inflammation after immunization and after low dose challenge (Fig. 2B). PepD treatment induced the highest inflammation in the heart as was observed in the joints.

**Figure 3:**
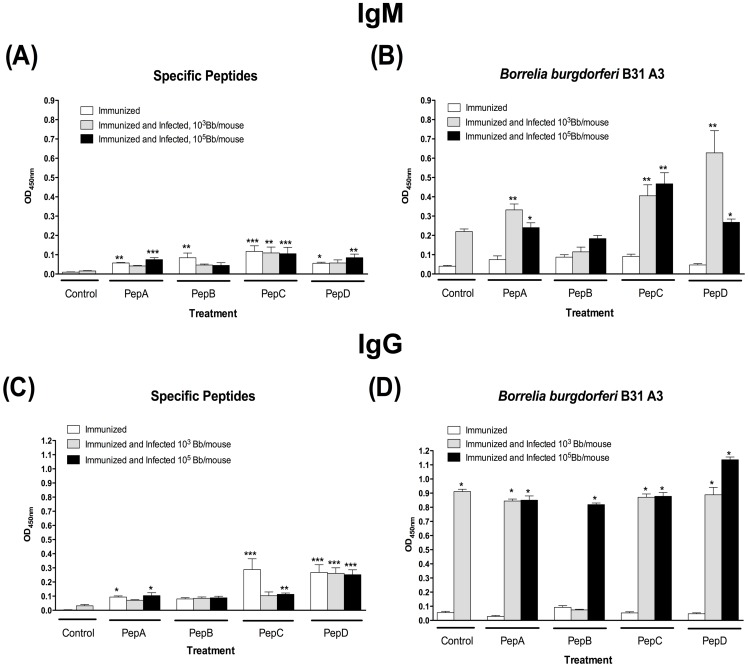
Low IgM and IgG antibodies were detected 4-weeks post priming in all groups. Antibody levels were evaluated 4-weeks post-priming as well as 4-weeks post needle infection. (**A**) Peptide-specific IgM antibodies. (**B**) *B. burgdorferi*-specific IgM antibodies. (**C**) Peptide-specific IgG antibodies. (**D**) *B. burgdorferi*-specific IgG antibodies. * Denotes statistically significant differences (* *P* value <0.05; ** *P* value < 0.01; *** *P* value < 0.001) when compared with the control group.

Evaluation of antibody responses showed that peptide specific IgG and IgM were relatively low in all groups regardless of the treatment received, with slight increase in antibody levels after immunization with peptides C and D (Fig. 3A and C). In addition, the presence of *B. burgdorferi* specific antibodies was very low in all groups after the immunization schedule was completed. Nevertheless, *B. burgdorferi* specific antibody titers (Fig. 3B and D) were significantly amplified in all groups after challenge infection, except for the IgG levels in the pepB vaccines challenged with the low borrelial dose. Moreover, serum cross-reactivity in between peptides was not observed (data not shown).

Since pepB induced the best protection, immunization and needle challenge was repeated two more times and similar results were observed with no recovery of bacteria from tissues of mice challenged with 10^3^ *Borrelia*/mouse. Consequently, pepB and pepD (which showed no protection) were selected for the subsequent studies using tick infection to evaluate protection (Table 1).

### Efficacy of pepB vaccine

**Figure 4:**
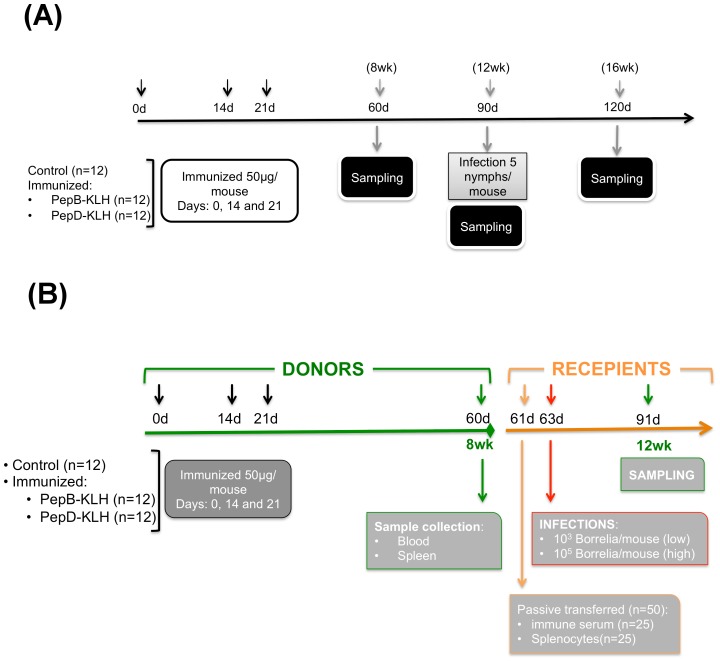
Summary of the study design. **(A)** Schematic representation of the efficacy study. C3H/HeN mice were immunized with peptide B or D derived from the VWFA domain of BB0172 conjugated to KLH and administered at 50 µg/mouse with equal volume of TiterMax® Gold (Sigma-Aldrich) at days 0, 14 and 21. Eight weeks post-priming, a subgroup of mice (4/treatment) were sampled to determine antibody levels and pathological side effects. Twelve weeks post-priming a second subgroup of mice (4/treatment) were euthanized and sampled for antibody levels in blood, T-cell activity (from draining lymph nodes and spleens) and tissue damage. At the same time, a final group of 4 mice/treatment was infected by tick challenge, utilizing 8 infected *Ixodes scapulars* nymphs/mouse (containing around 150 *Borrelia*/nymph). Sixteen weeks post-priming mice were euthanized and protection evaluated by determining bacterial recovery from tissues as well as bacterial burden, tissue damage and antibody levels in blood. **(B)** Schematic representation of the passive transfer experiment conducted during phase II. Donor C3H/HeN were immunized with peptide B or D administered at days 0, 14, and 21. Eight weeks post-priming, donor mice were euthanized and blood and spleens were collected. Serum and splenocytes were isolated and passively transferred to recipient mice. Two-days after transfer mice were infected with either a low (10^3^ spriochetes/mouse) or a high (10^5^ spirochetes/mouse) dose of *B. burgdorferi* B31 by subcutaneous inoculation. Four weeks post-challenge mice were euthanized and protection was evaluated.

**Figure 5:**
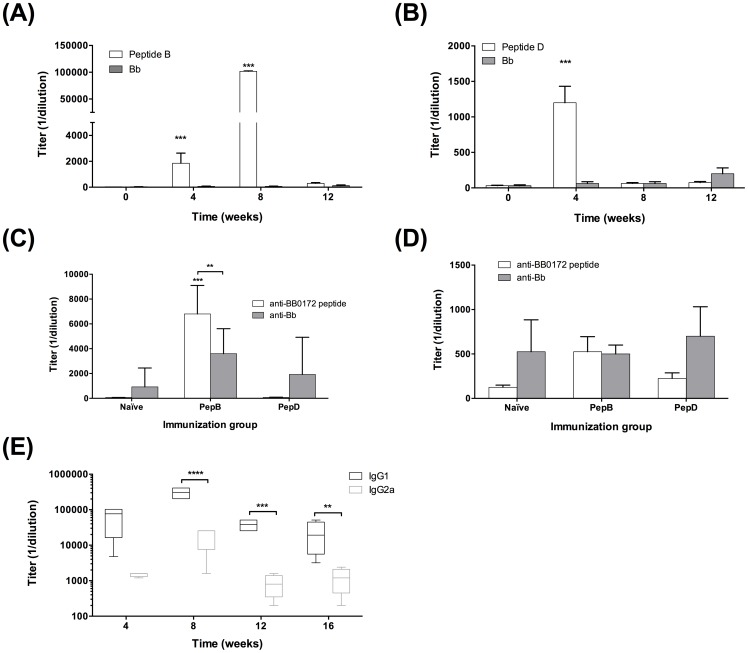
Peptide B-specific antibodies picked 8-weeks post immunization and were significantly stimulated 4 weeks post-tick infection. **(A)** IgG antibodies specific to Peptide B (open bars) and *B. burgdorferi* (grey bars) at weeks 0, 4, 8, and 12 post-priming. **(B)** IgG antibodies specific to Peptide D (open bars) and *B. burgdorferi* (grey bars) at weeks 0, 4, 8, and 12 post-priming. IgG **(C)** and IgM **(D)** antibody levels specific to each of the BB0172 peptide (open bars) and *B. burgdorferi* (gray bars) in control and animals immunized with either Peptide B or D 4-weeks post tick infection. **(E)** IgG1 and IgG2b antibody levels after 4, 8, 12 and 16 weeks post-priming with pepB. Titer represented in parenthesis. * Denotes statistically significant differences (* *P* value <0.05; ** *P* value < 0.01; *** *P* value < 0.001) when compared with the control group.

**BB0172 Peptide B – specific antibody titers peak 8-weeks post-priming.** Mice were immunized with pepB as described above (Fig. 4A) and blood samples for serum were collected at the time of priming as well as 4, 8, and 12-weeks post-priming. PepB-specific IgG antibodies peaked at 8-weeks post-priming and decreased to levels closer to basal at 12-weeks post-priming (Fig. 5A). The negative control mice immunized with peptide D had a small IgG antibody peak 4-weeks post-priming (Fig. 5B) and this outcome was similar to the result observed in the previous screening experiment (Fig. 3C). In addition, none of the serum samples from immunized mice reacted with *B. burgdorferi* whole cell lysates. Twelve weeks post-priming, the antibody levels had reduced to basal levels and mice were challenged by applying 5 *B. burgdorferi* infected ticks per mouse. Four weeks post-challenge, all the mice were euthanized and blood samples were collected. PepB-immunized mice had the highest peptide-specific IgG antibody levels as well as the anti-*B. burgdorferi* IgG levels (Fig. 5C). Notice that the antibody titers were also significantly higher in the pepB-immunized group starting at 4-weeks post-priming with maximum titers of 102,400 observed at 8-weeks post-priming (Fig. 5A). Four weeks post-tick challenge, pepB-immunized mice showed peptide-specific IgG titers of 6,800 significantly higher than those observed in naïve infected mice and pepD-immunized mice (Fig. 5C). Similar results were observed when *Bb*-specific IgG titers were evaluated. The pepB vaccinees had IgG antibody titers of 4,266 post-challenge, which was significantly higher than the titers observed in the naïve infected mice and the pepD vaccinees post-challenge mice (925 and 1,925 respectively). In addition, IgM antibody levels remained very low throughout this experiment (data not shown) as observed in the previous study (Fig. 3 A and B). PepD-specific IgM antibodies increased slightly after tick challenge (Fig. 5D), whereas pepB-specific IgM antibodies remained at basal level similar to the results observed in the earlier study (Fig. 3 A and B). Protective IgG antibody levels specific for pepB started peaking at 4-weeks post priming, reaching maximum levels at 8-weeks post-priming.

In addition, we measured the levels of IgG1 and IgG2a in the pepB-immunized group at 4, 8, and 12-weeks post-priming as well as at 4-weeks post-tick challenge (16 weeks). As observed in Fig. 5E, IgG1 titers were significantly higher than IgG2a with a peak at 12 weeks post-priming (307,200 and 78,400 respectively). Four weeks post-challenge, IgG1 titers (23,200) remained similar to those observed at 12-weeks post-priming (38,400) and were significantly higher than levels measured for IgG2a at the same time points (850, 12-weeks post-priming and 1,250 post-challenge). This observation suggests that the immune response after immunization and tick infection skewed towards Th2.

**Figure 6:**
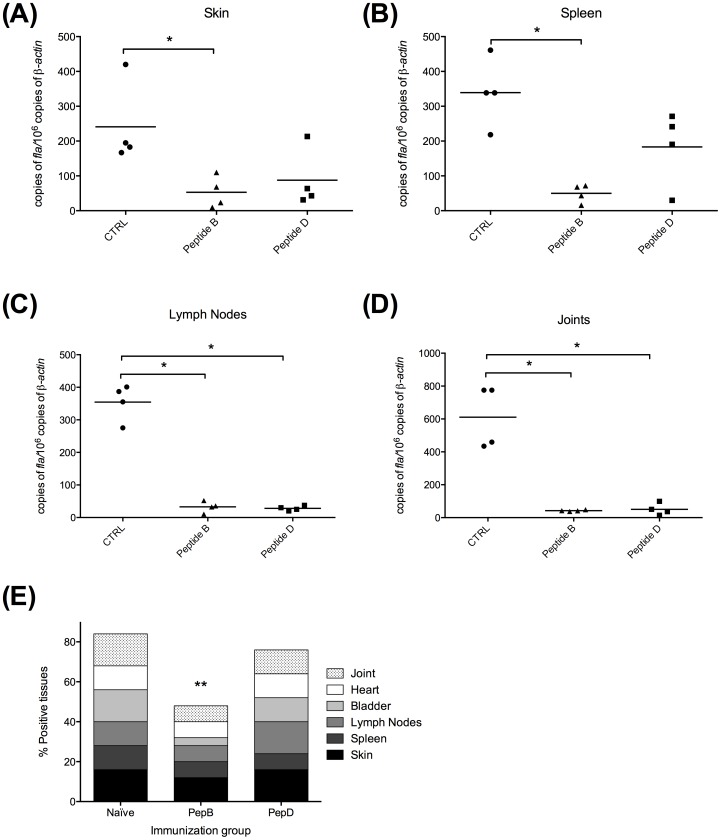
Peptide B induces partial protection in mice infected by using the tick model. Bacterial burden in tissues was significantly lower in animal immunized with Peptide B especially in skin (**A**) and spleen (**B**). Lymph nodes (**C**) and joints (**D**) show lower bacterial burden in both Peptide B and D immunized mice. Nevertheless, the bacterial recovery in cultures (**E**) was significantly reduced in mice receiving the Peptide B formulation compared with Peptide D or the control group. * Denotes statistically significant differences (* *P* value <0.05; ** *P* value < 0.01) when compared with the control group.

**BB0172 Peptide B partially protects against Lyme disease after tick challenge.** After tracking antibody responses in the mice immunized with pepB, the mice were challenged to determine whether or not the high antibody levels could protect mice against Lyme disease. At 12-weeks post-priming, mice were housed individually in wire bottom cages and challenged by applying 5 infected *I. scapularis* nymphs with an average of 100 *B. burgdorferi* cells per nymph. The mice were euthanized 4 weeks after ticks were applied. Analysis of skin, spleen, inguinal lymph node, bladder, heart and tibiotarsal joint tissues showed that pepB-immunized group had a significantly lower percent of positive cultures, compared with control and pepD-treated mice (Fig. 6E). Importantly, the outcome from this challenge study using the tick infection model, a 50% vaccine efficacy was achieved. In addition, the bladders of the pepB-immunized mice had less bacterial load when compared with the control and pepD-immunized groups (Fig. 6E). Furthermore, the bacterial burden in skin and spleens of mice immunized with pepB were significantly lower compared with the control group (Fig. 6 A and B). Lymph nodes and joints had very low bacterial burden in both immunized groups regardless of the peptide used (Fig. 6 C and D). Overall, the pepB-immunized mice had the lowest bacterial burden following challenge using infected ticks suggesting that this is a good candidate for the development of a Lyme disease vaccine of use in veterinary medicine.

**Figure 7:**
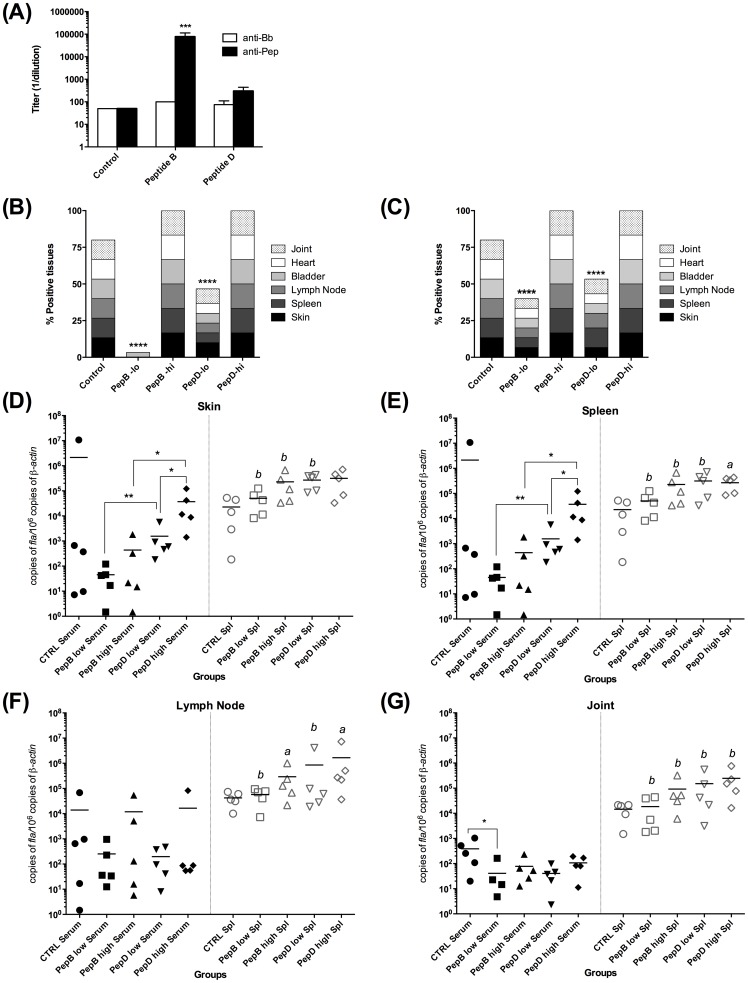
Peptide B-specific antibodies confer protection against *B. burgdorferi* infection. Antibody titers of control, peptide B-immunized, and peptide D-immunized animals. Open bars represent the anti-*B. burgdorferi* titers and black bars represent the peptide-specific antibody titers in each group (**A**). Bacterial recuperation from tissues of animals infected after passively transferring peptide specific serum (**B**) or splenocytes (**C**) to naïve mice. Bacterial burden was evaluated by qPCR in skin (**D**), spleen (**E**), lymph nodes (**F**), and joints (**G**). Bacterial recuperation from tissues and quantification was done 21 days-post infection. * Denotes statistically significant differences (* *P* value <0.05; ** *P* value < 0.01) when compared within the passive transfer treatment, while ***a*** (*P* value <0.05) and ***b*** (*P* value < 0.01) denote significant differences in between animals receiving serum or splenocytes from the same treatment (peptide B or peptide D).

**Peptide B-specific antibodies are responsible for protection against Lyme disease.** To determine whether the protection observed in the pepB-immunized mice was due to the high antibody titers or the cellular immune response (Fig. 4B), donor mice were immunized with pepB, pepD, or adjuvant only (control). When the peptide-specific antibody titers peaked at eight-weeks post-priming (Fig. 7A, peptide B: 100,000; peptide D: 300; control: 50) serum and splenocytes from each donor group were transferred to recipient mice, and then challenged 48 hours after transfer. PepB-specific antibodies protected mice challenged with low doses of *B. burgdorferi* B31 (10 × ID_50_) while no protection was observed in the other groups (Fig. 7B). In addition, splenocytes from pepB-immunized mice conferred partial protection, which suggests a protective role of splenocytes (Fig. 7C). Analysis of bacterial burden in different tissues of the recipient mice showed that animals that received anti-pepB specific antibodies had very low bacterial numbers in tissues, especially skin and spleen, compared to the control group or the anti-peptide D treated group (Fig. 7 D-G). The mice that received splenocytes had higher bacterial burden than those that received antiserum. Moreover, mice that received splenocytes from pepB vaccinees had the lowest bacterial burden in lymph nodes and joints, compared to those that received splenocytes from the control and pepD-immunized mice (Fig. 7 F and G). No difference in bacterial burden was observed between treatments in the skins and spleens. These results suggest the relevance of specific antibodies to block colonization.

**BB0172 peptide derived antigens are safe when injected subcutaneously in C3H/HeN mice.** The safety and tolerability of pepB immunogen was evaluated in C3H/HeN mice by histological evaluation of tissues at 4, 8, and 12 weeks post-inoculation. Most significant inflammation was mainly observed after infection with high bacterial doses, regardless of the vaccine candidate used (Fig. 2). Only minimal myocarditis and synovitis were observed in mice after immunization with pepB, as described above. Furthermore, no histological changes or areas of inflammation were observed in additional tissues evaluated at 8 and 12 weeks post-priming (skin, heart, tibiotarsal joint, liver and kidney, data not shown). Consequently, in this animal model, the BB0172 pepB antigen was shown to be particularly safe. Further studies in other animal models need to be done in order to confirm the safety of this vaccine candidate.

**Figure 8:**
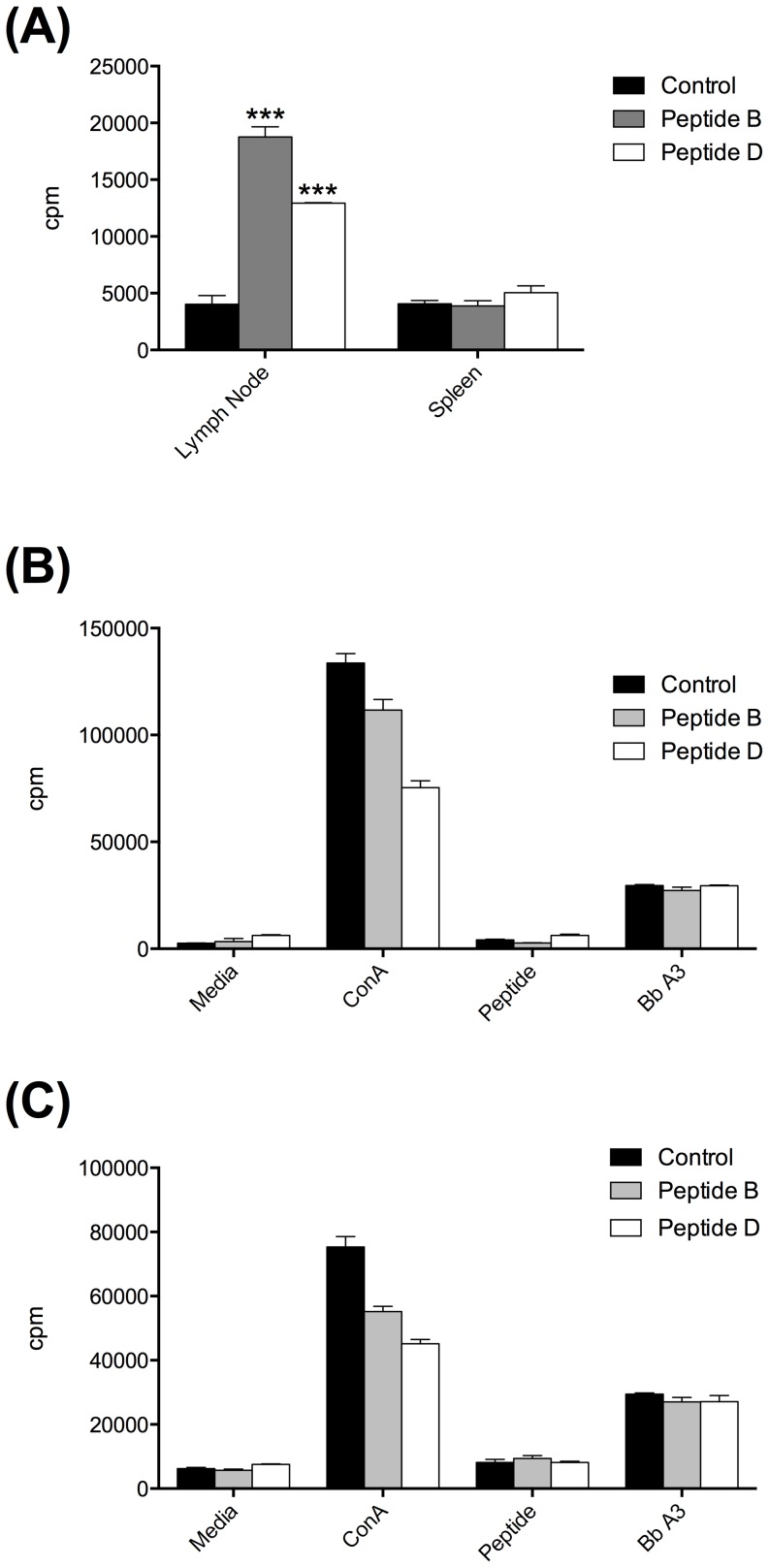
Proliferation assay of T-cells isolated from lymph nodes and spleens of mice immunized with peptide B (grey bars), peptide D (open bars) and controls (closed bars). **(A)** Proliferation assay at 8 weeks post-priming. Notice the high activity of cells isolated from lymph nodes of immunized compared with the control mice without any stimulation of the cultures. Proliferation assay of cell isolated from lymph nodes **(B)** and spleens **(C)** at 12 weeks post- priming. Concavalin A (ConA) was used as positive control for stimulation of the cell cultures. Specific peptides and *B. burgdorferi* B31 A3 strain whole cell lysates were used as test antigens to stimulate the cultures. Mean ± standard error of the mean, is represented for each lymphocyte proliferation measured. * Denotes statistically significant differences (* *P* value <0.05; ** *P* value < 0.01) when compared with the control group.

**T-cell response.** PepB-specific T-cell responses in mice immunized with the KLH-pepB conjugate or the control KLH-peptide D conjugate were tested by proliferation assays using cells isolated from lymph nodes or spleens. At 8 weeks post-priming, significant pepB-specific T-cell responses and pepD-specific T-cell responses were detected in the cells isolated from the lymph nodes draining the immunization sites but not in splenocytes (Fig. 8A). This outcome was rather unusual given that primed antigen-specific T-cells were also expected to be detected in the spleen. However, at 12 weeks post-priming, no pepB-, pepD-, nor *B. burgdorferi* B31 A3-specific T-cell responses were detected in the lymph nodes (Fig. 8B) or splenocytes (Fig. 8C). The cells from these tissues responded well to conA mitogen suggesting that the cells were healthy (Fig. 8 B and C).

## Discussion

Currently there is no commercial LD vaccine available in the market to protect humans, and hence we primarily rely on other preventive measures to control the incidence of this disease, particularly in endemic areas. A number of vaccine candidates have been studied and tested in the mouse model for Lyme disease as well as in wildlife [27]–[31] in an effort to control the spread of this disease. Most of the approaches used in the last few years are based on the outer-membrane lipoproteins OspA and OspC [32]–[37], together with a few novel antigens such as BBA52 [38], [39].

Dogs and horses have been identified as sentinels for Lyme disease across the US [56]–[58]. Under this scenario, and since Lyme disease affects both humans and companion animals, the development of a DIVA vaccine (differentiating infected from vaccinated animals) will be of great value in the control of Lyme disease utilizing a global health approach [59]–[62]. The DIVA vaccine strategy will not only improve our diagnostic capabilities, but also helps us in the prevention of Lyme disease in companion animals and in the reduction of reservoir competence. Therefore, a new Lyme DIVA vaccine can significantly impact the prevention of LD in humans and animals.

Our previous studies have identified BB0172, a chromosomally encoded borrelial protein anchored to the outer membrane through two hydrophobic domains [48]. In addition, BB0172 is conditionally expressed and has been shown to bind to integrins α3β1 *in vitro* as it is relatively conserved among *Borrelia* species [48]. Therefore, we hypothesized that this protein could be an effective vaccine candidate due to both its function as an adhesin and the fact that sera from naturally infected animals did not react to this protein in ELISA and immunoblot assays [48]. Consequently, we developed a number of short peptides conjugated to a hapten (KLH). Our results showed that mice immunized with the pepB formulation were protected against infection with pathogenic *B. burgdorferi* administered by injection at low infectious doses. These results supported the hypothesis that pepB could be a strong vaccine candidate to prevent Lyme disease. In addition, no inflammation was observed in hearts and joints from animals receiving this vaccine formulation, even after infecting with low doses of *B. burgdorferi*. Very low peptide-specific antibody titers were observed in this first screening experiment. After infection, only high *B. burgdorferi* specific antibody titers were generated in all groups except in animals immunized with pepB and infected with low borrelial doses.

Following low dose *B. burgdorferi* challenge, pepB conferred the highest vaccine efficacy (100%) compared with the other peptides tested, and therefore was selected as a potential DIVA vaccine antigen. We also selected pepD as a negative control as it is a peptide from the same protein but it did not confer protection. In our studies, the antibody titers for pepB consistently increased during the weeks following the immunization schedule, peaking at 8-weeks post-priming. We evaluated the protection acquired after vaccination by exposing the mice to *B. burgdorferi* through the natural route of infection. Using infected *I. scapularis* ticks directly after the antibody levels returned to basal level, we consistently observed that pepB-immunized mice were significantly protected against infection.

Passive transfer of sera from pepB-immunized, but not from pepD-immunized mice, to naïve recipients conferred protection upon challenge. This suggested that antibodies play a role in protection, an outcome that is consistent with previous demonstrations that anti-*B. burgdorferi* antibodies play a significant role in protection [63]–[67]. Analysis of pepB-specific antibody isotypes revealed IgG1 dominance, suggesting a Th2-type immune response, which was consistent with previous findings [68]–[70]. Adoptive transfer of splenocytes from pepB-immunized, but not from pepD-immunized mice, conferred partial protection. This outcome could have been due, in part, to the presence of pepB-specific antibodies secreted by memory B-cells in splenocytes. If the presence of memory B-cells was responsible for the partial protection, it is not clear why the cells did not undergo recall upon challenge, but it was notable that no pepB-specific T-cells were detected in spleens by proliferation assay. In addition, pepB-specific splenocytes were transferred to recipient mice through intravenous administration while *B. burgdorferi* was administered by subcutaneous needle injection. The discrepancy in administration of both splenocytes and the infectious agent could explain why the B-cells injected did not generate enough antibodies to neutralize *B. burgdorferi* after infection. Under these circumstances, B-cells will tend to migrate to the spleen while the borrelial cells will prefer the draining lymph nodes, skin and joints [4], [71], [72]. The disparity in tissue tropisms may account for the discrepancy in the results observed, where passive transfer of pepB-specific antibodies induce protection, and the transfer of pepB-specific splenocytes did not [73]. Additional studies are needed to define the role played by T-cells in protection.

Further studies need to be done in order to improve the protection, mostly by improving the delivery method as well as the hapten/adjuvant with which this antigen is administrated. In particular, delivery of the vaccine antigen utilizing viral particles [74], [75], as well as the use of microneedles [76]–[79] for the delivery of vaccines can significantly improve the immune response and consequently protection after both needle and tick infection. In addition, by using transdermal inoculation we will be stimulating the cell types that most likely will be the encountered by the bacterium after the tick bite [78], [79].

Taken together, an improved DIVA vaccine will significantly impact the prevention and control of Lyme diseases as well as its surveillance since it will be compatible with currently available tests for the detection of Lyme diseases in animals such as IFA, ELISA and immunoblot assay (in particular, the C6 base technology (IDEXX laboratories Inc)), without the necessity of developing further tests to detect infected animals. With the vaccine antigen pepB, regular ELISA tests can differentiate which animals have been vaccinated (react to pepB antigen only) from those that have been infected (react to *B. burgdorferi* extract only), and also those that had received the vaccine and are undergoing infection (react to both pepB and *B. burgdorferi* extract in ELISA), making pepB a suitable candidate for the development of a DIVA vaccine.

## Materials and Methods

### Ethics statement

All animal experiments were done following the Texas A&M University IACUC approved animal use protocol #2010-124. Texas A&M has adopted the “U.S. Government Principles for the Utilization and Care of Vertebrate Animals Used in Testing, Research and Training,” and complies with all applicable federal, state, and local laws which impact the care and use of animals.

### Identification of potential vaccine target peptides from BB0172 antigen

***Borrelia burgdorferi*** **strains and growing conditions.** *B. burgdorferi* B31 A3 (*Bb*) virulent isolate was used throughout this study. In order to obtain an antigenic profile similar to that observed in the natural infection, we grew this bacterium at room temperature (RT) and pH 7.6 to mimic the unfed tick conditions. Once the cultures reached a cell density of 1–2×10^7^ spirochetes/ml a subculture was transferred to 37°C, 1% CO_2_, and pH 6.8 mimicking the conditions in the tick upon feeding. To run the ELISA tests using whole cell lysates, *B. burgdorferi* was grown in 500 ml cultures shifted from RT/pH 7.6 to 37°C/pH 6.8 and 1% CO_2_. After cultures reached a cell density of 3–5×10^7^ spirochetes/ml, cells were harvested, washed three times with HBSS buffer (HyClone, Thermo Scientific Inc.), quantified, and lysed using 0.1 mm glass beads in 2 ml screw cap tubes in a BeadRuptor 24 (Omni International, Inc). After the lysis cycle, the glass beads were sedimented by quick centrifugation and the supernatants were stored at –20°C in 1 ml aliquots until use in the ELISA assays. For the needle infection experiments, *Bb* cultures were similarly prepared. The bacterial cultures were shifted from RT/pH 7.6 to 37°C/pH 6.8 and reaching a density of 3–5×10^7^ spirochetes/ml prior to being harvested, washed three times with HBSS buffer, and re-suspended in HBSS containing inactivated normal rabbit serum (50:50, *v:v*). The cultures were then quantified and diluted to the appropriate cell density (10^3^ or 10^5^ spirochetes/ml).

**Peptide design.** The BB0172 antigen is a *B. burgdorferi* membrane protein which is poorly immunogenic in the murine model of Lyme disease [48]. Four peptides within the vWFA domain of BB0172 were designed considering their probability of being exposed to the external environment and distance from a potential internal glycosylation site. The peptides have been designated by the letters A through D (pepA, pepB, pepC and pepD). Peptides were synthetized at Peptide 2.0 Inc. (Chantilly, VA) at 98% purity and conjugated to Keyhole Limpet Hemocyanin (KLH) to ensure immunogenicity. The same peptides were synthetized without conjugation to KLH for *in vitro* T-cell and ELISA assays.

**Immunization protocol.** The protective immunity elicited by each one of the KLH-conjugated BB0172 peptides (A, B, C or D), was evaluated in mice. Groups of 6-8 week old female C3H/HeN mice (n = 12) were inoculated subcutaneously with each one of the KLH-conjugated peptide at a dose of 50 µg/mouse formulated in TiterMax® Gold (*v:v*, Sigma-Aldrich, St. Louis, MO) at days 0, 14 and 21 (Fig. 1). A group of six mice similarly inoculated with adjuvant alone served as the negative controls. One week after the last boost and prior to challenge, four mice per group were euthanized and sampled to evaluate the antibody levels in serum and T-cell proliferation in draining lymph nodes and spleens. Samples from the heart and tibiotarsal joint from these mice were evaluated histologically to rule out possible side effects due to the antigen administration. Mice were infected by needle inoculation one week after the last boost as described below.

***B. burgdorferi*** **challenge protocols.** To determine which peptide elicited protection in the murine model of Lyme disease, four mice per immunized group were challenged by subcutaneous needle inoculation with 10^3^ (low) or 10^5^ (high) *Bb*/mouse 28 days post-priming as described above (Fig. 1). The challenge doses used correspond to 10× and 1000× the infectious dose-50 (ID_50_), respectively. Control mice were infected with only one dose, 10^3^ spirochetes/mouse (10× ID_50_).

To evaluate protection, the mice were euthanized 28 days post-challenge and blood samples were collected to evaluate antibody levels. Skin, spleen, inguinal lymph nodes, heart, bladder and tibiotarsal joint were collected from each mouse for bacterial recovery in BSK-II media complemented with 6% inactivated normal rabbit serum and incubated at 32°C and 1% CO_2_. Five days post inoculation cultures were blind passed to prevent inhibition of bacterial growth by tissue degradation. Blind passaged cultures were incubated at 32°C and 1% CO_2_ for 15 days before evaluating bacterial growth by dark field microscopy [49]. One piece of heart and a tibiotarsal joint were collected for histopathology. Finally, a piece of skin, a small piece of spleen, one inguinal lymph node and one joint were collected for evaluation of bacterial burden by qPCR as previously described [50]. All animal experiments were conducted following the Institutional Animal Care and Use Committee and the Biosafety committee recommendations.

**Histopathology.** Mouse tissues were collected 4-weeks post-priming and 4 weeks after challenge as described above. Tissues were fixed in 10% buffered formalin, processed for routine histopathology, paraffin embedded, sectioned and stained with H&E. The tibiotarsal bones and joints were decalcified in 10% EDTA prior to being processed for histopathology. A board certified pathologist blindly evaluated all tissues. Inflammation in selected tissues were scored from 0–4 based on the following scale: normal  =  0 (no inflammation), minimal  = 1 (one small foci of inflammation), mild  =  2 (2–5 foci of inflammation with increased numbers of inflammatory cells), moderate  =  3 (multifocal inflammation with significant number of inflammatory cells), and severe  = 4 (multifocal to diffuse, with more than 30% of section infiltrated with inflammatory cells) [51].

**Enzyme linked Immuno-sorbent Assay.** Sera from immunized mice (0, and 4 weeks post-priming) as well as from animals immunized and then challenged (4 weeks post-challenge) were evaluated for IgG and IgM levels by ELISA. 96-well MaxiSorb® plates (Nunc, Thermo Scientific, Ltd.) were coated overnight at 4°C with either 500 ng/well of each one of the BB0172 peptides or with the whole cell lysate of *B. burgdorferi* A3 strain (10^7^ Borrelia/well) grown at RT/pH7.6 and shifted to 37°C/pH 6.8 as described above. Carbonate buffer pH 9.1 was used for coating the ELISA plates and after coating, the plates were washed three times in Phosphate Buffered Saline (PBS) containing 0.2% Tween 20 (PBS-T) and blocked for 2 hours at room temperature in PBS-T containing 3% Bovine Serum Albumin (BSA). Blocked plates were washed three times in PBS-T and mouse serum samples were added in duplicates and in 2-fold serial dilutions ranging from 1:100 to 1:102,400 in PBS-T containing 1% BSA. Plates were incubated for 1 hour at room temperature and unbound primary antibodies were removed by washing plates three times in PBS-T. Secondary anti-mouse HRP conjugated antibody was added to the plates at 1:3000 dilution in PBS-T containing 1% BSA. After washing, plates were incubated with OPD (*o*-phenylenediamine dihydrochloride) color substrate following manufacturer recommendations (Pierce, Thermo Scientific, Ltd). After a 20-minute incubation in the dark, plates were read at a wavelength of 450 nm and analyzed using the BMI LABTECH OMEGA plate reader and software. All samples were evaluated in triplicates.

### Efficacy of pepB vaccine

***B. burgdorferi*** **growing conditions.** *Bb* B31 A3 virulent isolate was also used throughout this section of the study. Culture conditions were the same as described above. In addition, *Bb* used for *in vitro* infection of *Ixodes scapularis* nymphs was grown in BSK-II media pH 7.6 and 1% CO_2_ until cultures reached a cell density of 2×10^7^ spirochetes/ml.

**Immunization protocol.** The same immunization protocol as described in the target identification phase above was used in the efficacy study (Fig. 4A). PepB was used to immunized mice (n = 12) since it was the only peptide that conferred protection in the target identification phase. PepD (n = 12) served as an internal negative control since it did not confer protection and in addition, a control group receiving adjuvant only was also included (n = 12). Vaccine safety was evaluated at 8 and 12 weeks post-priming (Fig. 4A). Protection was evaluated 12-weeks post-priming following challenge using *Bb*-infected ticks (Fig. 4A). Four-weeks post-challenge, the mice were euthanized and protection and safety were evaluated as described below.

**Passive transfer protocol.** To evaluate the role of antibodies and lymphocytes in the protection induced against Lyme disease in mice immunized with pepB, we conducted passive transfer studies in which groups of donor mice (control, pepB and pepD) were immunized following the immunization protocol described above (Fig. 4B). Twelve weeks post-priming, the mice were euthanized, and blood and spleens were collected. Splenocytes were isolated from each of the groups as well as serum following procedures described elsewhere [52], and pooled splenocytes and serum were passively transferred to recipient mice (Fig. 4B). Recipient mice were divided into 6 groups (n = 10). Three groups were inoculated with 300 µl/mouse of serum samples from control, pepB or pepD-immunized mice, whereas the other three groups were similarly inoculated but with 4×10^7^splenocytes/mouse from control, pepB, or pepD-immunized mice, respectively. Forty-eight hours post-transfer, all the mice were challenged by needle inoculation with either a low or a high dose of *B. burgdorferi* as described below. Four weeks post-infection, the mice were euthanized and protection evaluated (Fig. 4B).

***B. burgdorferi*** **challenge protocols.** The protection elicited by the peptides B and D in the murine model of Lyme disease was evaluated by challenging the mice with infected *I. scapularis* ticks (Fig. 4A) 12-weeks post-priming. To conduct this study, naïve *I. scapularis* nymphs were purchased from the Oklahoma State University Tick Laboratory. Nymphs were desiccated for 4 days at 79% relative humidity (RH) in a chamber, then were dipped in a suspension of 10^8^ spirochetes/ml for 45 minutes. After the 45 minute infection, the ticks were washed and placed in the same 79% RH chamber for 3 days in order to improve attachment of the nymphs to mice [53]. Prior to the challenge, a group of 10 ticks was used to evaluate the level of infection with *B. burgdorferi* by quantitative real time PCR (qPCR). Immunized C3H/HeN mice were infested with 5 infected nymphs per mouse and housed in wire bottom cages following standard operational procedures. Ticks were left to feed on mice until repletion.

The challenge protocol described in the target identification phase was used to evaluate the protection conferred by passive transfer of specific serum or adoptive transfer of splenocytes (Fig. 4B). In both needle and tick challenge, mice were euthanized 28 days post-infection and blood samples were collected to evaluate antibody levels. Skin, spleen, inguinal lymph nodes, heart, bladder and tibiotarsal joint were collected from each mouse for bacterial recovery in BSK-II media as previously described [49]. One ear, a piece of liver, one kidney, a piece of heart and a tibiotarsal joint were collected for histopathology. Finally, a piece of skin, a small piece of spleen, one inguinal lymph node and one joint were collected for evaluation of bacterial burden by qPCR as previously described [50].

**Enzyme linked Immuno-sorbent Assay.** Sera from immunized mice (0, 4, 8, and 12 weeks post-priming) as well as from animals immunized and then challenged (4 weeks post-challenge) were evaluated for IgG and IgM levels by ELISA as described above.

**Histopathology.** Mouse tissues were collected after immunization (4, 8, and 12 weeks post-priming) and 4 weeks after challenge as described above. Tissues were processed in the same way as described in the target identification phase above. A board certified pathologist blindly evaluated all tissues, and inflammation in selected tissues was scored from 0–4 as described above.

**T-cell proliferation assay.** Priming of *Bb* pepB-specific T-cell responses was tested by proliferation assays using cells isolated from the lymph nodes or spleens as previously described [54], [55]. Two months post-immunization, single cell suspensions were isolated from pooled lymph nodes or spleens from four mice immunized with the KLH-pepB conjugate or from three control mice. Proliferation assay was conducted using 5×10^5^ cells/well in triplicate-wells of 96-well plates in a total volume of 100 µl of complete medium containing different doses of pepB (0.01, 0.1, 1, 2.5, 5, or 10 µg/ml). The positive control was 1.25 µg/ml concanavalin A (conA), whereas medium alone served as a negative control. In addition, whole cell lysates of *B. burgdorferi* B31 A3 isolate was included in this assay (serial dilutions as above). The cells were cultured for 72 hours at 37°C with 5% CO_2_ then labeled with 0.25 µCi of ^3^H-thymidine for 6 hours, collected using an automated cell harvester (Tomtec). The incorporated ^3^H-thymidine was counted with a liquid scintillation counter. The incorporation of ^3^H-thymidine by the proliferating lymphocytes was presented as mean counts per minute (cpm) of triplicate wells.

In a second experiment, cells were isolated from the lymph nodes and spleens from mice 3 months post-immunization with the KLH-pepB conjugate and proliferations assays were conducted as above. Naïve mice and mice immunized with KLH-pepD conjugate served as controls. The positive control was 1.25 µg/ml conA, whereas medium alone served as a negative control. In addition, whole cell lysates of *B. burgdorferi* B31 A3 isolate was also included in this assay. The cultures were labeled and processed as above.

### Statistics

Bacterial recovery from tissues was analyzed using the Two-way ANOVA to determine significant differences in between treatments. Quantitative real time PCR data were analyzed using the Mann Whitney U test to determine differences in the bacterial burden determined in each group compared with the control group. In addition, antibody levels were also analyzed utilizing a Two-way ANOVA with the Bonferroni multiple comparison test, in which all groups were compared to the control group. All tests and graphics were performed using Prism 6.0d (GraphPad Software, Inc.).
